# Applying Dialectical Behavior Therapy as a Transdiagnostic Treatment in a Case of Borderline Personality Disorder and Eating Disorder

**DOI:** 10.1002/jclp.23754

**Published:** 2024-11-26

**Authors:** María Vicenta Navarro‐Haro, Alba Abanades Morillo, Azucena García‐Palacios

**Affiliations:** ^1^ Department of Psychology and Sociology, Faculty of Social and Human Science University of Zaragoza Teruel Spain; ^2^ Biomedic Research Center of Aragon (CIBA) Instituto de Investigación Sanitaria Aragón Zaragoza Spain; ^3^ Department of Psychology and Sociology Faculty of Social and Human Sciences, University Jaume I Castellón de la Plana Spain

**Keywords:** borderline personality disorder, case study, Dialectical Behavior Therapy, eating disorder, emotion dysregulation, transdiagnostic treatment

## Abstract

This article presents a case study of a 31‐year‐old woman with a dual diagnosis of Borderline Personality Disorder (BPD) and Eating Disorder Not Otherwise Specified (EDNOS). Paula received a 12‐month Dialectical Behavior Therapy (DBT) outpatient treatment. DBT is considered a transdiagnostic treatment approach to address emotion dysregulation, which shifts the therapy focus traditionally placed only on behavioral change toward including also validation and acceptance and dialectical strategies. DBT addresses eating symptomatology as a dysfunctional form of emotional regulation and has shown promising results regarding its efficacy for the treatment of BPD and EDNOS comorbidity. Given the growing evidence, a standard DBT treatment plan was developed for this case. Specifically, pretreatment and phase 1 of the DBT program are described. During pretreatment and phase 1, individual therapy aims to improve and maintain client's motivation to change and engage in treatment, as well as to establish and prioritize treatment goals. As for group therapy, the main goal of the skills training in DBT is to enhance individual's capability by increasing skillful behavior (mindfulness, emotion regulation, distress tolerance, and interpersonal effectiveness skills). Paula received 24 weekly skills training sessions over a year. The results after a 12‐month standard DBT treatment showed that Paula no longer met criteria for BPD, she had a significant decrease in difficulties in emotional regulation and impulsiveness and in EDNOS symptomatology. This case study may enhance learning about how to apply a transdiagnostic treatment to address BPD and EDNOS together in clinical practice.

## 1 Introduction

This article aims to present the treatment based on Dialectical Behavior Therapy (Linehan 1993, 2015) applied in a clinical case (for more information see García‐Palacios and Navarro‐Haro 2023) with a diagnosis of Borderline Personality Disorder (BPD) and Eating Disorder Not Otherwise Specified (EDNOS). DBT was originally designed for the cognitive‐behavioral treatment of people with BPD and suicidal behaviors (Linehan 1987). However, it is considered currently an example of the evolution of behavior therapy because it includes innovations aimed at providing an adequate response to a very complex problem, persistent emotional dysregulation. One of the most innovative aspects of DBT is a change of focus in therapy. Classical cognitive‐behavioral therapy focuses on achieving the resolution of emotional problems through behavioral and cognitive change. However, DBT emphasizes acceptance and validation strategies, to achieve change from there. The ultimate goal of DBT is that the person not only learns to survive, but to have a “life worth living,” with meaning.

Linehan developed DBT considering a biosocial theory, which advocates that BPD's maladaptive behaviors (including eating dysfunctional behaviors) are considered as failed attempts to regulate emotions, a consequence of the interaction between a biological emotional vulnerability and an invalidating environment (Crowell, Beauchaine, and Linehan 2009; Linehan 1993). DBT treatment focuses on shaping and reinforcing more adaptive behaviors to improve emotion regulation while providing patients with a validating environment. DBT strategies are based on principles of dialectical philosophy, behavior therapy, and Zen practice. The main strategies are *validation* of the person's capacities, so that the patient learns to trust and validate their own intimate experiences, as well as *problem solving* strategies to modulate extreme emotionality and, thus, reduce dysfunctional behavior. Group skills training is a key component of therapy, which emphasizes the balance between acceptance skills (mindfulness and distress tolerance) and change skills (emotional regulation and interpersonal effectiveness). DBT has four intervention formats (individual psychotherapy, group skills training, telephone coaching, and consultation team meetings for therapists) which aim to work on the following functions respectively: (1) to improve client's motivation to change and engage in treatment; (2) to enhance individual's capability by increasing skillful behavior; (3) to ensure generalization of change and restructuring or changing the individual's environment; (4) to enhance therapist motivation to deliver effective treatment (Linehan 2015). DBT is also organized by different phases and goals of treatment. Table 1 describes the phases and goals of the treatment program (for more information, see Linehan 1993, 2015).

**Table 1:** Phases and goals of the treatment program.

Phases	Treatment goals
Pretreatment	General objective: Therapy commitments and agreements
Phase 1: Severe behavioral dyscontrol	General objective: Stability and behavioral control
	Decrease of suicidal behaviors: Life‐threatening symptoms such as suicidal thinking, suicidal behavior and self‐harm
	Decrease any behaviors that interfere with therapy working. Decrease of behaviors that interfere severely with one's quality of life.
	Increase of behavioral skills: Mindfulness, emotional regulation, distress tolerance, interpersonal efficacy
Phase 2: Quiet desperation	General objective: Experiencing emotions following a pattern of experiential avoidance
	Reduce posttraumatic stress/pathological grief
	Decrease residual disorders
Phase 3: Living problems/less severe disorders	General objective: Ordinary happiness and unhappiness
	Increasing self‐respect and achieving individual goals
Phase 4: Feeling incomplete	General objective: Freedom and enjoyment
	Reduce feelings of emptiness and loneliness and increase feelings of fulfillment

*Source:* Adapted from Linehan (1993, 2015).

For all these reasons, DBT is considered a transdiagnostic intervention (Linehan, Bohus, and Lynch 2007) and a third‐generation therapy (Hayes 2004). In this sense, some researchers have studied the efficacy of DBT in other disorders with difficulties in emotion regulation. Some adaptations have been developed to treat problems comorbid to BPD, such as substance use disorders or eating disorders. Regarding the results of the scientific efficacy, according to the latest systematic reviews (e.g., Storebø et al. 2020), DBT provides a larger number of controlled clinical trials (compared to other interventions that have shown efficacy) supporting the evidence of its suitability to treat BPD.

Considering BPD and eating disorders comorbidity, Fairburn, Cooper and Shafran (2003) proposed a transdiagnostic theory of maintenance mechanisms shared among eating disorders (e.g., clinical perfectionism, mood intolerance) after finding common migration among eating disorder (ED) symptoms long‐term. Fairburn et al. clinically observed that extreme dietary restraint was prominent in most atypical eating disorders (EDNOS) and that it was sometimes accompanied by self‐induced vomiting, over‐exercising, or laxative misuse. Binge eating was also frequent and, in most cases, over‐evaluation of eating, shape and weight and their control was observed too, hallmarks of the two prototypic eating disorders (anorexia and bulimia nervosa). In a 10‐year follow‐up study of the course of comorbid EDs in people diagnosed with BPD, it was found that diagnostic migration of EDs was also common long term and that new onsets of EDNOS were higher (around 40%) than new onsets of other more specific eating disorders during the follow‐up (Zanarini et al. 2010). These findings suggest that migration trajectories of comorbid EDs are similar in people diagnosed with BPD.

In terms of research evidence, studies indicate a high prevalence of comorbid BPD and ED symptoms in samples with ED, including interpersonal difficulties, unstable self‐image, marked impulsivity, and emotion dysregulation (e.g., Martinussen et al. 2017; Newton 2019). A conceptual review suggested that both anorexia and bulimia nervosa are characterized by emotion regulation deficits (e.g., low repertoire of adaptive emotion regulation skills and increased use of maladaptive skills; Lavender et al. 2015).

Given the shared etiological factors (e.g., emotion dysregulation) in individuals with co‐occurrent BPD and ED, transdiagnostic treatment approaches treating emotion regulation, such as Dialectical Behavior Therapy, have been recommended as an adequate treatment to address BPD and ED psychopathology (e.g., Liakopoulou et al. 2023) In DBT treatment, ED dysfunctional behaviors (e.g., purging, bingeing, etc.) are conceptualized as an attempt to regulate unwanted emotions (e.g., Safer, Telch, and Agras 2001). The most recent American Psychiatric Association guideline (APA 2023) also recommends targeting certain personality traits, such as perfectionism, emotional instability, impulsiveness, and interpersonal difficulties, when treating EDs. There is also preliminary research evidence of DBT to treat BPD and ED comorbidity (e.g., Liakopoulou et al. 2023; Navarro‐Haro et al. 2018, 2021) as well as eating disorders only (specially for binge eating disorder and bulimia nervosa; see Ben‐Porath et al. [2020] for a review).

## 2 Case Illustration

### 2.1 Presenting Problem and Client Description

Paula is a 31‐year‐old single woman with higher education, although she has never worked as a professional. She is the youngest of two sisters. The patient lives with her mother. Her father works in another city and he is only at home on weekends. Her sister also lives in another city and they do not have a close relationship.

Regarding personal history, Paula reports feeling abandoned by her father when he got a job abroad and the family had to move to another city for a while. She explains that it was very difficult to adapt to the new city, especially in the school environment. It took her about 2 years to integrate into the new school since she had a lot of insecurities and complexes about her physical appearance. The mother explains that Paula has reported dissatisfaction with her physical appearance since she was an adolescent. However, she has had more stable periods and was able to finish her university studies. Her parents acknowledge that there has been a lack of boundaries with Paula since childhood. The father presents a history of anxious‐depressive symptomatology and the mother informed of food restriction and anxiety symptoms during the assessment.

Concerning clinical history, Paula has presented dysfunctional eating behaviors since childhood. She started psychological and psychiatric treatment when she was 14 years old for anorexia nervosa symptoms and finished it when she gained weight. At the age of 17, she began another treatment for anxiety and depression. At 23, she was hospitalized for specific treatment for her eating disorder and left a few months later. At the age of 25, she was hospitalized due to an emotional crisis and depressive symptoms after being isolated at home for 6 months. Subsequently, she was diagnosed with BPD and began non‐DBT treatment during 6 months, but dropped out because she did not feel capable of attending therapy. A year later, she was hospitalized for a month at the same hospital to stabilize the medication and reduce binge eating. A few years later, she took a medication overdose, which she refers to as a suicide attempt (it was unplanned, ambivalent and impulsive) and went to the emergency room. When she went back home, she refers to several episodes of self‐harm. One of them was burning herself with a cigarette and scratching her head. This last episode of self‐injury was accompanied by suicidal ideation. At the end of that year, the problems worsened due to the time spent in isolation at home, ending with verbal and physical aggression toward her mother, at which time she asked for help and started DBT treatment.

When Paula comes to therapy, daily functioning is very altered. Paula is not able to fend for herself, she finds it difficult to attend therapy for fear of going out due to body dissatisfaction. In addition, she has no social relationships and only maintains telephone contact with a friend. She has not seen her sister for a year. The relationship with her mother is conflictive. There are continuous arguments with her father, as she does not feel understood by him. She finished her degree but has not felt capable of working afterward. She did some social volunteering but ended up quitting.

Paula is unable to attend therapy on her own due to emotional instability and distorted self‐image (she avoids leaving the house for fear of being watched). Paula arrives at the center in great distress, as her weight has increased due to binge eating. She decided to ask for help to treat binge eating specifically and after several episodes of verbal and physical aggression toward her mother. She agrees to give DBT treatment a chance. She had previously received a few months of therapy (with another therapist and another treatment) at our center and reported that she did not feel better. Paula explains that the treatments for the eating disorder made her problem worse, as prolonged hospital admissions increased her feelings of hopelessness. She is motivated to start therapy, although she is not confident that it will solve her problem. She is willing to treat the BPD but she is reluctant to treat the eating problem as a whole: motivated to work on reducing binge eating, but not to decrease food restriction since it helps her lose weight, which makes her feel better in the short term.

After the usual assessment in the personality disorders unit (see Table 3 for the battery of instruments applied and results of the assessment), Paula was diagnosed with BPD and an Eating Disorder Not Otherwise Specified (EDNOS) based on DSM‐IV‐TR.

Regarding the BPD pattern, Paula presents emotional instability, with periods of severe depressive symptomatology (anhedonia, bedridden, excessive crying, hopelessness) and euphoria. In addition, she shows irritability and angry outbursts with her immediate family, especially when she is criticized or contradicted. Behaviorally, she is verbally aggressive toward her parents and occasionally physically aggressive toward her mother (associated with anger attacks). She reports previous self‐injurious behaviors and a suicide attempt that are not present in the last 6 months. On the cognitive level, she reports self‐invalidating thoughts and distrust of others and hopelessness. She also presents suicidal ideation.

Concerning the EDNOS, Paula presents a severe pattern of dysfunctional eating behaviors daily (3–5 binge‐eating episodes per week in the last month) that are compensated with some restrictions (skipping meals, normally dinner, every day in the last month). She does not have compensatory behaviors (vomiting, laxatives, or exercise). She also reports frequent body image distortion and weight and body checking behaviors (twice per day in the last month) and clothing checks (e.g., checking if clothes fit), rituals and ruminations regarding body and health as well as excessive perfectionism.

### 2.2 The Therapist

Therapist is a 40‐year‐old woman. She is a clinical psychologist with 10 years of experience working in the transdiagnostic treatment of emotional disorders, including borderline personality disorder and different comorbidities such as eating disorders. She is also a DBT certified clinician by Linehan Board of Certification (DBT‐LBC, Certified Clinician) and DBT trainer by Behavioral Tech Institute since 2018.

### 2.3 Case Formulation

As explained above, from the biosocial theory (Crowell, Beauchaine, and Linehan 2009; M. Linehan 1993), maladaptive behaviors (including dysfunctional eating behaviors) are considered a problem of *emotion dysregulation*, which is the result of a reciprocal interaction between a biological *emotional vulnerability* of the patient and an *invalidating environment* over time. The emotion dysregulation over time also increases the risk of psychopathology in different areas. Linehan proposed five areas of dysregulation: behavior dysregulation, emotion dysregulation, cognitive dysregulation, and identity disturbance (see Crowell, Beauchaine, and Linehan 2009). Below is a summary of Paula's case formulation, explained according to the biosocial theory. It includes the main etiological factors as well as the symptomatology of Paula when she started the treatment in the five dysregulation areas.

#### 2.3.1 Emotional Vulnerability

It is characterized by three fundamental aspects: (1) Emotional sensitivity: For example, anguish since childhood if her father did not praise her for an achievement. (2) Emotional intensity: For example, high anger when the mother tried to give her food that she did not want; (3) Slow return to emotional baseline: Difficulty modulating anger and anxiety.

#### 2.3.2 Invalidating Environment/Other Environmental Problems

In this case, the environment was characterized by a lack of structure and boundaries and emotional expression inhibition. Her parents did not follow or enforce meal and bedtime schedules. Her parents did not have an intimate relationship between them and were not affectionate with Paula (she felt little emotional attention). Her father did not praise achievements (e.g., good grades in class) and oversimplified her emotional difficulties during childhood (“it's no big deal”). Her mother did not recognize that she herself had also issues with food (meal restriction, sneaky eating). Her father left home to work in another city and Paula felt abandoned. Paula also suffered verbal bullying during high school.

#### 2.3.3 Transaction Between Vulnerability and Disabling Environment

An example of transaction between Paula's vulnerability and the environment: Paula feels sad and asks her parents to buy her unhealthy food; if they do not buy it, she reacts with anger and starts yelling at them and insulting them. When that happens, her parents agree to buy her the food, which positively reinforces verbally aggressive behavior.

#### 2.3.4 Paula's Psychopathology According to Five Areas of Dysregulation

1. Behavioral dysregulation: Binge eating; food restriction; avoidance behaviors (e.g., not going out if feeling anxious); body checking behaviors (e.g., weighing oneself several times a day); cigarette smoking.
2. Interpersonal dysregulation: Fear of abandonment by her father; verbal aggression toward her parents and physical aggression toward her mother; social isolation (stays at home all day, no friends).
3. Identity dysregulation: Distortion of self‐image; feelings of emptiness; self‐identification as mentally ill.
4. Cognitive dysregulation: Overvalued idea about body image and weight (e.g., I am fat); polarized thinking (e.g., either I am the best in therapy or in class, or I am stupid); hopelessness (e.g., I'm not going to get better); self‐criticism (e.g., I'm fat, I disgust myself).
5. Emotional dysregulation: Difficulties regulating anger; persistent emotional instability (depression, anger, euphoria); major depressive episodes.

### 2.4 Course of Treatment

The treatment program Paula received was standard Dialectical Behavioral Therapy (Linehan 1993, 2015) in the outpatient personality disorders unit of a private hospital. The treatment consisted of a combination of individual and group interventions that were carried out during phase 1 of DBT, over 12 months of treatment, usually the minimum required to initiate DBT. In parallel to receiving individual and group therapy, she was offered the possibility of using telephone calls with the therapist to generalize the skills learned in session and to learn to manage distress in times of crisis. The therapist also attended consultation team meetings weekly to maintain adherence to the treatment and receive support from the team. Paula's treatment goals, as well as the main strategies used in individual and group therapy, are described below.

#### 2.4.1 Individual Therapy

The individual treatment consisted of one 60‐min session per week with a therapist specialized in personality disorders and certified in DBT. The main goal of individual therapy is to improve and maintain the client's motivation to change and engage in treatment (Linehan 2015). As previously discussed, standard DBT has a series of phases. In this article, the pretreatment and phase 1 of the DBT strategies used with Paula will be explained.

During pretreatment, therapy agreements as well as individual goals and treatment goals are raised. The treatment goals are hierarchized and addressed according to their importance. The hierarchy established for intervention in individual therapy is as follows: (1) Reducing life‐threatening behaviors; (2) Reducing therapy‐interfering behaviors; (3) Reducing quality of life interfering behaviors; (4) Increasing behavioral skills (usually learned in group therapy).

Therefore, the specific therapy objectives for each patient are organized according to this hierarchy of priorities. The personal therapy goals and DBT treatment goals agreed upon with Paula in pretreatment are described below. Some of the personal goals were agreed during pretreatment DBT and others were continued to be worked on in phase 1.

##### 2.4.1.1 Personal Goals

To decrease: Verbal aggression toward her parents; resentment towards her father for leaving home and moving to another city (she felt abandoned by her father); binge eating; body obsession and body control; self‐judgment and self‐criticism.

To Increase: Acceptance of her parents' functioning; acceptance of her body; perception of self‐efficacy and self‐esteem; social interactions and enjoyable activities; independent behaviors (e.g., taking the train to therapy).

##### 2.4.1.2 DBT Treatment Goals

1. Decrease life‐threatening behaviors: Few current reasons for living, previous suicidal ideation; previous self‐harm (e.g., medication overdose, burning oneself with a cigarette). Although some of these behaviors were not occurring when she started, they were assessed during treatment.
2. Decrease therapy‐interfering behaviors: Difficulty attending therapy; failure to follow various recommendations of the therapist and team (e.g., receiving ancillary treatment for the eating disorder such as going to the day hospital or taking a specific medication because it might increase weight); making negative comments about the effectiveness of the therapy.
3. Decrease behaviors that interfere with quality of life: Dysfunctional eating behaviors (binge eating, skipping meals); distortion of self‐image; verbal aggression during crises; rigid thinking and rumination; mood‐dependent behaviors; social isolation; low activity level.

In the illustration of a session below, the main strategies used during individual therapy are described.

###### 2.4.1.2.1 Group Skills Training

The main goal of skills training in DBT is to enhance the individual's capability by increasing skillful behavior. As recommended in standard DBT, Paula received 24 weekly skills training sessions that were repeated twice during the year, so the skills training lasted approximately 12 months. The duration of the skills training session was around 2 h and it was conducted in a group format (8–10 patients in total). The group session was conducted by a lead therapist and a co‐therapist, both trained in DBT. The group therapy was composed of four modules, two of them to practice the acceptance component (mindfulness and distress tolerance) and the other two to work on change skills (emotional regulation and interpersonal effectiveness). Table 2 shows the skills training program that Paula received.

**Table 2:** Contents of DBT group skills training.

Modules	Week	Sessions
Mindfulness	1	OR/M‐Session 1. Orientation and mindfulness “what” skills
	2	M‐Session 2. Mindfulness “how” skills
Disstress tolerance	3	DT‐Session 3. Crisis survival
	4	DT‐Session 4. Crisis survival
	5	DT‐Session 5. Crisis survival
	6	DT‐Session 6. Acceptance of reality
	7	DT‐Session 7. Acceptance of reality
	8	DT‐Session 8. Acceptance of reality
Mindfulness	9	M‐Session 9. Orientation and mindfulness “what” skills
	10	M‐Session 10. Mindfulness “how” skills
Emotion regulation	11	ER‐Session 11. Objectives of the emotion regulation model and understanding emotions
	12	ER‐Session 12. Changing the emotions
	13	ER‐Session 13. Changing the emotions
	14	ER‐Session 14. Changing the emotions
	15	ER‐Session 15. Reducing vulnerability
	16	ER‐Session 16. Reducing vulnerability
	17	ER‐Session 17. Reducing vulnerability
Mindfulness	18	M‐Session 18. Orientation and mindfulness “what” skills
	19	M‐Session 19. Mindfulness “how” skills
Interpersonal effectiveness	20	IE‐Session 20. Objetives IE
	21	IE‐ Session 21. Learning to achieve our objectives (DEARMAN)
	22	IE‐Session 22. Learning to maintain the relationship (GIVE)
	23	IE‐ Session 23. Learning to maintain self‐respect (FAST)
	24	IE‐ Session 24. Evaluate options for saying no and asking questions

Regarding the goal of increasing coping skills, the specific therapy objectives that were agreed with Paula at pretreatment were the following:

− Mindfulness. Mindfulness of thoughts, one‐mindfully skill practice and participation.
− Distress tolerance: Survival crisis skills before displaying problem behavior, radical acceptance of parents' behavior and present difficulties to engage in social relationships.
− Interpersonal efficacy: DEARMAN assertiveness skills to negotiate with her parents and GIVE to learn to respect them and maintain a good relationship with them.
− Emotional regulation: Identifying emotions and performing opposite actions to the emotion and practicing problem solving.

#### 2.4.2 Illustration of an Individual Therapy Session

Individual therapy session 10 of phase 1 has been selected to illustrate DBT strategies employed in this case. In the same way that it is recommended in standard DBT (Linehan 1993), the main strategies used in individual therapy to reduce dysfunctional behaviors and increase problem solving were the following:

##### 2.4.2.1 Review the DBT Diary Card and Conduct a Behavioral Analysis of Problem Behavior Associated With the Hierarchy of Treatment Goals

**Figure 1:**
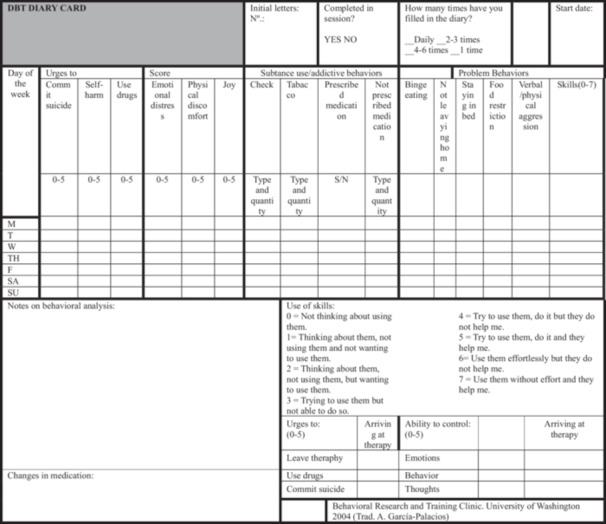
Diary prepared for Paula.

After reviewing the diary card (Figure 1 shows the diary developed for Paula) and following hierarchy of treatment goals, a chain analysis is normally performed to analyze the patient's dysfunctional behaviors.

**Figure 2:**
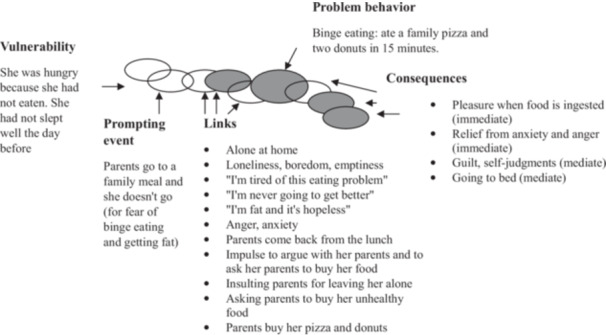
Chain analysis of binge eating episode.

In this session, an episode of binge eating carried out by Paula on a Wednesday was selected to conduct a chain analysis. The following is the chain analysis of Paula's binge eating behavior, taking into account the DBT model. Figure 2 shows an outline of the analysis carried out in session 10.

The main control variables of the chain analysis that were worked on during the session were the following:

*Vulnerability*: Failure to follow structured sleep and meal times.

*Dysfunctional links:*

− Emotions: difficulties in tolerating emptiness, loneliness, and anger.
− Cognitions: Hopelessness “she would never get better” (getting better means being very thin), “she will always be fat, not worthy, and people will not like her.”
− Behaviors: asking parents (aggressively) to buy her food to binge eat by justifying that she could not control it.

*Consequences*: Pleasure and immediate relief (positive and negative reinforcement) short term, and isolation and self‐judgment long term.

##### 2.4.2.2 Conduct a Solution Analysis: Problem Solving Related to Behavioral Analysis

After the chain analysis was conducted, a series of DBT strategies were carried out during the session and a solution analysis was conducted. Different solutions were agreed upon that day in therapy to address problem behavior and the control factors. Below the DBT strategies (Linehan 1993) used during the therapy session are explained. Some transcripts of the therapist talking are included.

Validation strategies: The therapist validated Paula's difficulty in tolerating loneliness and the frustration of not feeling capable of leaving the house to go to the lunch.

Therapist: “Paula, it makes sense that you felt lonely when your parents left and frustrated for not being able to go to your uncle's lunch. I understand that you would have been worried about binge eating if you had gone to the lunch.”

Contingency clarification: The therapist explained to Paula how the problem behavior is reinforced and helped her, through the chain analysis, to be aware that binge eating and verbal aggression toward her parents are not effective strategies to solve the problem long term.

Therapist: “As you can see in the chain, after you asked your parents aggressively to buy you unhealthy food, your parents accepted it and it led you to access the food and, therefore, binge eating. After eating pizza and donuts, you felt some pleasure and relief from the anxiety and anger. It is clear that these behaviors worked to reduce distress short term, but do you think yelling at your parents and binge eating would help you to achieve your therapy goals, such as being able to go out and have social interactions, long term?”

Dialectical strategies: Dialectical techniques were used to work on rigid and extreme thinking such as: finding a synthesis between the poles of going or not going to the lunch or making lemonade out of lemons after staying at home.

Therapist: “When you are talking to me about what happened on Wednesday, I can identify two opposite sides: either you stayed at home or you went to your uncles' lunch and binge ate. I wonder if we could find a middle path between these two opposites. It comes to my mind the idea of you going to lunch with your uncles and bringing your own food from home. What do you think? Nonetheless, staying at home alone could be also a very good opportunity to practice skills.”

##### 2.4.2.3 Skills Generalization: Practicing Techniques Learned in Therapy

At the end of the session, different skills (Linehan 2015) were proposed to work on different aspects of the chain analysis:

Mindfulness skills: Observing the present moment skill for ruminating thoughts about future limitations that do not exist, and body scan meditation to learn to accept the body.

Distress tolerance skills: Practicing crisis survival skills for high intensity of anger: change temperature (ice pack), distraction, use crisis survival kit.

Interpersonal effectiveness skills: practice assertiveness using DEAR MAN skill for asking her parents to stay with her during meals and GIVE skill to validate her parent's need to socialize with other family members, as well as repairing problem behavior (e.g., insulting them).

Reality acceptance skills: Radical acceptance of difficulty tolerating family events involving food until she improves her eating problem; reality acceptance of her body: practicing willing hands and half‐smile skills in the session.

Emotional regulation skills: Practicing opposite action skill to anger: instead of insulting her parents, trying to put herself in their place; using problem solving skill: going to the family meal with her own food; practicing skills to reduce negative vulnerability; improving bedtimes: no naps and going to bed earlier; eating meals at agreed‐upon times and engaging in pleasant activities to increase self‐efficacy.

#### 2.4.3 Ancillary Treatments

Paula received biweekly psychiatric visits. One of the main problems during pretreatment was Paula's difficulty in attending therapy. As a solution at the beginning of therapy, she was offered support to drive her to the center for about 2 months. This solution improved adherence to treatment long term. However, the patient missed several months of therapy due to depressive symptoms, especially during the Christmas and summer periods: at Christmas because of increased binge eating and fear of leaving the house due to body image distortion, and in summer due to having to wear less clothes, which increased her body image distortion.

She was also going to day‐hospital a few hours each day during 3 months to decrease isolation and increase functioning at the beginning of therapy. She was referred to a nutritionist to motivate her to increase her calorie intake during periods of restriction and to eat healthy food and structure mealtimes, although she decided to abandon sessions with the nutritionist after a month.

Paula was working on healthy eating habits and strategies to reduce binge eating, such as monitoring of food every day. In addition, contingency management (e.g., storing clothes in small sizes and not using the mirror at home) was used to reduce clothing and body checking. To decrease image distortion, exposure to the body in therapy was used. A focus on loving kindness and self‐compassion was included during the exposure.

### 2.5 Outcome and Prognosis

During the assessment phase, semi‐structured interviews and self‐reports were conducted to assess the personality disorder and associated symptomatology. In addition, the DBT diary was used to assess Paula's level of distress and frequency on specific problem behaviors once a week. Table 3 describes the variables assessed and the assessment instruments and scores obtained at baseline and after 1 year of DBT treatment.

**Table 3:** Scores on the assessment instruments at baseline and after 1 year of treatment.

Variable	Instruments	Baseline (pretest)	After a year of treatment (posttest)
Interviews			
Diagnosis of personality disorders	The Structured Clinical Interview for DSM‐IV Personality Disorders, SCID‐II, First et al. (1996)	Avoidant PD = 3^a^	Avoidant PD = 0
		Borderline PD = 5^a^	Borderline PD = 3
		Histrionic PD = 4	Histrionic PD = 0
		Antisocial PD = 3	Antisocial PD = 0
		Obsessive‐compulsive PD = 3	Obsessive‐compulsive PD = 1.
		Paranoid PD = 3	Paranoid PD = 1
Diagnosis of BPD	Diagnostic Interview for Borderlines‐Revised, validated by Barrachina et al. (2004)	Affect scale = 1	Total score = 1 (does not meet the criteria)
		Cognition scale = 1	
		Impulse Action Pattern scale = 2	
		Interpersonal relationships scale = 2	
		Total score = 6 (cut‐off score to meet criteria is 6)	
Severity of BPD	Clinical global impression of BPD (ICGTLP, Perez et al. 2007)	Total severity = 4 (moderate)	Overall improvement = 2 (greatly improved)
Self‐reports			
Difficulties in emotional regulation	DERS, Difficulties in Emotion Regulation Scale, validated by Hervás and Jódar (2008)	Total score = 80 (significant scores)	Total score = 67 (normative scores: 58.4)
Trait impulsiveness	BIS11, Barratt Impulsiveness Scale, validated by Oquendo et al. (2001)	Total score = 40 (significant scores)	Total score = 32 (normative score: 32.5)
Depressive symptoms	Inventario de Depresión de Beck, versión II (BDI‐II, Beck Depression Inventory, validated by Sanz et al. (2003)	Total score = 17 (mild symptomatology)	Total score = 15 (absent symptomatology)
State and trait anxiety	STAI, State‐Trait Anxiety Inventory, validated by Spielberger et al. (1982)	State Anxiety = 20 (50th centile)	State anxiety = 20 (50th centile)
		Trait Anxiety = 45 (85th centile)	Trait Anxiety = 35 (75th centile)

^a^ Meets criteria for the personality disorder.

As can be seen in Table 3, after 1 year of treatment, Paula no longer meets the criteria for any of the personality disorders, nor for BPD. In addition, there has been a significant reduction (scores close to normality) in emotional regulation difficulties, impulsivity, trait anxiety, and BPD severity. At the behavioral and functional level, several improvements are observed, such as reduction of verbal aggression, and attending therapy more frequently and she has also initiated social interactions with a former friend and has signed up for French classes.

Despite the improvement, Paula still engages in several dysfunctional behaviors (e.g., sporadic verbal aggression) and there are some therapy‐interfering behaviors (e.g., difficulty attending therapy alone, not following some recommendations regarding her eating problems) that we need to work on before moving to phase 2. As can been seen in the behavioral analysis, parental reinforcement of the eating pattern was detected during therapy since her parents buy Paula the unhealthy food, which led to binge eating and further periods of food restriction. Paula also ate at different times from her family and a special meal was prepared for her. Her parents were undergoing family treatment and joint therapies were carried out to work on eating habits at home and interpersonal relationships.

Specifically, for the EDNOS and related problems, Paula has significantly decreased binge eating (twice in the last month, with mild severity) and continues being at normal weight. Although it has improved, Paula still carries out some food restriction (skipping dinner, 3 days per week, 12 times in the last month). The weight and clothing checks have decreased considerably (2 days per week, eight times in the last month). Ruminating thinking, perfectionism and image distortion have not decreased significantly.

Concerning the skills practice, she has begun using interpersonal effectiveness skills with her parents and practicing crisis survival and mindfulness skills. However, Paula still has difficulty understanding and generalizing the skills in her daily life, for example, applying crisis coping skills before problem behavior and using phone coaching in crisis moments.

## 3 Clinical Practices and Summary

This case study shows an example of applying DBT for the treatment of BPD and EDNOS comorbidity. Considering shared etiological factors (e.g., emotion dysregulation) in individuals with co‐occurrent BPD and ED, transdiagnostic treatment approaches treating emotion regulation, such as Dialectical Behavior Therapy, have been recommended as an adequate treatment to address BPD and ED psychopathology.

In this case, remission of BPD symptoms, emotion regulation and impulsiveness improvement were achieved during the first year of treatment. As can be seen in Table 1, phase 1 of DBT is focused on increasing stability and behavioral control. Results of this case study are consistent with phase 1 goal since the main impulsive behaviors were decreased. These findings are also as expected according to the scientific literature. In longitudinal studies, it has been observed that individuals with BPD show a significant reduction in symptoms, however, the improvement in general functioning is moderate (e.g., Temes and Zanarini 2018).

Furthermore, a decrease in some of the EDNOS‐related behaviors (binge eating, food restriction and weight and body checking) was also observed. However, other symptoms such as ruminating thinking, image distortion and perfectionism were not significantly reduced. As recommended in DBT (Linehan 1993), to improve resistant eating symptoms (e.g., perfectionism and image distortion) as well as social and academic/work adjustment, it would be necessary to continue to address phase 2 and 3 therapy goals with a focus on reducing emotional avoidance and improving social functioning.

During phase 1, auxiliary treatment strategies such as healthy eating habits, monitoring of food, contingency management, and exposure to the body were added to DBT standard to address EDNOS symptomology. However, since BPD was considered the primary diagnosis, no previously developed evidence‐based DBT for ED adaptations were used in this case (see Ben‐Porath et al. 2020 for a review). Considering the lack of improvement of some ED symptoms more related with anorexia nervosa (i.e., image distortion and perfectionism), it would be interesting to apply specific treatments such as Radically Opened DBT (RO‐DBT; Lynch 2018) which has been designed to address perfectionistic overcontrolled coping through teaching flexibility, openness, and social connectedness and has shown good preliminary evidence to improve anorexia nervosa (Little and Codd 2020; Lynch et al. 2013). Using self‐report measures to evaluated EDNOS related outcomes are also recommended for future studies.

To conclude, evidence shows high prevalence of comorbid BPD and ED personality problems (interpersonal difficulties, unstable self‐image, marked impulsivity, and emotion dysregulation) in people with ED, however, traditional ED treatments normally fail to include strategies to treat these problems. This case study may help inform of specific strategies to address BPD and EDNOS transdiagnostic problems (i.e., emotion dysregulation and impulsiveness) together in clinical practice. For future research, it may be relevant to refine treatment programs to address more resistant aspects such as perfectionism and image distortion.

## Ethics Statement

The authors declare that all ethical guidelines have been followed during the development of this article and appropriate consent was obtained. The procedures of this study were approved by the Clinical Research Ethics Committee of IdcSalud of Catalunya (reference number: 2017/05‐PSQ‐HGC).

## Conflicts of Interest

The authors declare no conflicts of interest.

## Data Availability

The data that support the findings of this study are available on request from the corresponding author. The data are not publicly available due to privacy or ethical restrictions.
